# Patterns of comorbidity and disease characteristics among patients with ankylosing spondylitis—a cross-sectional study

**DOI:** 10.1007/s10067-017-3894-0

**Published:** 2017-11-08

**Authors:** Lotta Ljung, Björn Sundström, Johan Smeds, Maria Ketonen, Helena Forsblad-d’Elia

**Affiliations:** Department of Public Health and Clinical Medicine/Rheumatology, Umeå University, S-901 87 Umeå, Sweden

**Keywords:** Ankylosing spondylitis, Comorbidity, Cross-sectional, Epidemiology

## Abstract

The knowledge of the development of comorbidities in patients with ankylosing spondylitis (AS) is limited. The aim of this study was to analyse associations between AS disease characteristics and comorbidity and to evaluate patterns of comorbidities in patients with AS. Patients with AS, fulfilling the modified New York Criteria, were identified (*n* = 346, mean age 56 ± 15 years, 75% men, 99% HLA B27 positive). Through a review of the patient records, data on disease activity parameters, laboratory results, disease manifestations, and diagnoses of any clinically significant comorbidity was obtained. Four categories of comorbidities of interest were identified: A. arrhythmias, conduction disorders, and valvular heart disease; B. atherosclerosis and atherosclerotic CVD; C. spinal and non-spinal fractures; and D. obstructive sleep apnoea syndrome. Associations between AS disease characteristics and comorbidities in categories were assessed in logistic regression models. Differences in proportions of comorbidities was analysed using two-sided chi-square. Age was associated with all four categories of comorbidities, and male sex with arrhythmias, conduction disorders, valvular heart disease, and obstructive sleep apnoea syndrome. Early disease onset and long disease duration, respectively, were associated with arrhythmias, conduction disorders, and valvular heart disease. Obstructive sleep apnoea syndrome was associated with features of the metabolic syndrome. Patients with atherosclerotic cardiovascular disease had an increased risk of most other comorbidities, similar to, but more pronounced than patients with arrhythmias, conduction disorders and valvular heart disease. Comorbid conditions motivate clinical awareness among patients with AS. Longitudinal studies are needed to establish preventive measures.

**Electronic supplementary material:**

The online version of this article (10.1007/s10067-017-3894-0) contains supplementary material, which is available to authorized users.

## Introduction

Ankylosing spondylitis (AS) is a chronic inflammatory disease, primarily involving the axial skeleton and entheses [1]. The disease is usually clinically manifested in the third decade of life, and men are overrepresented among patients with a diagnosis of AS [1]. The prevalence of AS mirrors the prevalence of the genetic factor, the MHC class I molecule HLA B27 in the population. In Sweden, this can be depicted by the south-north gradient and an observed prevalence of AS around 0.15% in the south and 0.25% in the northern parts of the country [2]. The clinical characteristics of AS, such as the risk of progressive spinal stiffness, are well known, but the total burden of the disease, taking also comorbidity into account, has been less studied. Some comorbidities, like anterior uveitis, inflammatory bowel disease, and psoriasis, might be considered as phenotypic features of the AS disease as such [3, 4]. This might also be the case for aortic regurgitation and cardiac conduction system disorders, and possibly also osteoporosis and spinal fractures, which all are related to the disease [5–10]. Other comorbidities observed to be associated with AS include hypertension, dyslipidaemia, diabetes, and sleep apnoea syndrome [3, 11–13]. An increased mortality risk, mainly attributed to cardiovascular disease (CVD), infections, and cervical spinal fractures, has been reported in AS [14–16]. In order to improve the clinical care for patients with this potentially debilitating and life-threatening disease, it is important to identify the risk factors for the development of the comorbid conditions as well as the relationships between comorbidities and the disease characteristics of the patients. In addition, it is also of interest to reveal if the comorbidities are related to each other.

The aim of this study was to evaluate comorbidities in a clinical population of patients with AS according to the modified New York Criteria [17], taking the phenotype of the AS disease into consideration. Firstly, the report will focus on four categories of comorbidities: A. arrhythmias, conduction disorders, and valvular heart disease; B. atherosclerosis and atherosclerotic CVD; C. spinal and non-spinal fractures; and D. obstructive sleep apnoea syndrome (OSAS), in relation to characteristics of the AS patients. Secondly, the presence of these particular comorbidities will be related to other comorbidities in order to reveal patterns of comorbidities in the AS patients.

## Methods

Västerbotten County in northern Sweden, with a population of 265,000 inhabitants, has one public clinic of rheumatology located at the university hospital with units in two other cities, and no private practitioners. Patients with AS treated with conventional synthetic or biologic disease modifying antirheumatic drugs (csDMARDs or bDMARDs) are followed by rheumatologists. Other patients with AS are offered yearly follow-up by physiotherapists, although for less severe disease the intervals between visits can be increased. Through the digital system of patient records, covering all three rheumatologic units in Västerbotten County, all individuals with a visit with a diagnosis of AS (ICD-10 M45.9) from a visit at the clinic of rheumatology between May 2002 (when the patient records were digitized) and November 2015 (*n* = 523) were identified. Individuals not fulfilling the modified New York Criteria [17] were excluded, leaving 346 patient files for thorough evaluation according to a pre-set form. Demographic data (age, sex, educational level, smoking habits); disease specific data (age at symptom onset, extra-articular disease manifestations, C-reactive protein (CRP) levels (g/L); mobility measurements, HLA-B27 status, treatment with csDMARDs or bDMARDs, glucocorticoids or nonsteroid anti-inflammatory drugs—NSAIDs); and coincidence of comorbidity (supplementary Table 1) were collected from all visits at the clinic of rheumatology. The evaluation period covered May 2002 to the end of December 2015. Data on comorbidities and events from other medical specialities including primary care was also collected through overviews of diagnoses from digital patient records covering all in- and out-patient care in the county, except out-patient care by approximately ten primary care units. The data on events from the records’ overview was complemented with data from the patient records, radiology reports, and other laboratory data if it was considered relevant and was available.

A comorbidity or an event was considered prevalent for a patient if ever occurring during the period May 2002–December 2015 or noted in any patient record as occurring at a time point antedating May 2002. The same consideration was made regarding smoking, and disease characteristics such as a description of arthritis, enthesitis, anterior uveitis, inflammatory bowel disease, or psoriasis in the patient record. Treatment with DMARDs, glucocorticoids, NSAIDs, or tumour necrosis factor inhibitors (TNFi) were registered if usage was noted in the patient record during or before the evaluation period. Mobility measurements from the latest recorded visit were used for calculation of an index for spinal mobility, based on the categories of mobility for each of five measurements defined in Bath Ankylosing Spondylitis Measurement Index (BASMI) [18]. Only for one patient, five spinal mobility measurement variables were available. Seventy-five individuals (21.7%) lacked all measurements, and 146 (42.2%) had four available variables. The remaining 124 (35.9%) patients had 1–3 variables. The mean score from all available measurements for each individual was included and is referred to as spinal mobility.

All patients but 18 (5.2%) had at least one analysed CRP value during the evaluation period. For patients with more than one CRP measurement, a mean of all available values was used. Missing data was also observed regarding the age at or date for disease onset (10 individuals, 2.9%); HLA B27 status (93 individuals, 26.9%); and smoking habits (132 individuals, 38.2%).

In this report, we choose to focus on four categories of comorbidities of interest in AS namely: A. valvular heart disease and/or arrhythmia, B. atherosclerotic CVD, C. spinal and non-spinal fractures, and D. OSAS. Group A included any valvular heart disease, atrial fibrillation, atrial flutter, and other arrhythmias or conduction disorders (Supplementary Table 2). Group B included ischemic heart disease, cerebrovascular disease, peripheral vascular disease, and congestive heart disease (Supplementary Table 2).

The study was performed in accordance with the Helsinki Declaration and was approved by the Ethical Review Board at Umeå University, Umeå, Sweden. The Ethics Review Board waived the requirement for individual consent for this retrospective, observational study.

### Statistical methods

Simple logistic regression models, adjusted for age at end of the evaluation period (or the age at death) and sex (if appropriate), were used to evaluate the associations between AS disease characteristics using the four main categories of comorbidities of interest (A–D), respectively, as dependent variables. The results are presented as odds ratios (ORs) with 95% confidence intervals (CI), and *p* value. In dichotomous variables, presence/history of a characteristic was coded 1 and no presence/history of a characteristic was coded 0. Male sex was coded 1 and female 2. Patients with missing data regarding the age at symptom onset, the mean of CRP, or spinal mobility were not included in the respective regression model. Patterns of comorbidities were graphically presented, with statistical significance of differences in proportions of comorbidities compared between the patients with and without the specific comorbidity of interest (A–D) analysed using two-sided chi-square. The level of significance was set at *p* < 0.05. All analyses were performed using IBM SPSS Statistics Version 24 for Mac.

## Results

Among 346 included patients with AS, one in four were female (Table 1). The reported disease onset occurred at a mean of 25 (standard deviation; SD 9) years of age, most patients were HLA B27 positive, and a majority of the patients were or had been smokers (Table 1). The mean disease duration, years (SD) at the end of the evaluation period (or death) was 31 (14) years, ranging from 1 to 60 years. Twenty-three patients died during the evaluation period, at a mean age (SD) of 73 (7) years. Disease characteristics of the included patients with AS are presented in Table 1.

**Table 1:** Characteristics of included patients with ankylosing spondylitis. Figures are numbers (%) unless stated otherwise

	All, *n* = 346
Female sex	85 (25)
Mean of age at disease onset, years (SD)*	25 (9)
Mean of age at the end of the evaluation period, years (SD)	56 (15)
Disease duration at the end of the evaluation period, years (SD)*	31 (14)
HLA B27 positivity*	250 (99)
Ever smoker*	126 (59)
Mean of CRP, mg/L (SD)*	11 (10)
Mean of spinal mobility (SD)*	4.5 (2.0)
csDMARD treated ever	128 (37)
TNFi treated ever	49 (14)
Glucocorticoid treated ever	106 (31)
NSAID treated ever	312 (90)
Peripheral arthritis	163 (47)
Enthesitis	180 (52)
Anterior uveitis	134 (39)
Inflammatory bowel disease	24 (7)
Psoriasis	11 (3)

*CRP* C-reactive protein, *csDMARD* conventional synthetic disease modifying antirheumatic drug, *TNFi* tumour necrosis factor inhibitor, *NSAID* nonsteroid anti-inflammatory drug

*Number of missing: age at disease onset = 10 (2.9%), HLA B27 = 93 (26.9%), smoking habits = 132 (38.2%), mean of CRP = 18 (5.2%), spinal mobility = 75 (21.7%)

### Frequencies of the main comorbidities of interest

Arrhythmias were registered for 34 patients (9.8%) and included mainly atrial fibrillation or flutter (*n* = 31). Other arrhythmias or conduction disorders (pacemaker, *n* = 1; pacemaker and atrioventricular block, second degree, *n* = 1; paroxysmal supraventricular tachycardia, *n* = 2; long QT syndrome, *n* = 1; left bundle branch block and atrioventricular block, first degree, *n* = 1; unspecified arrhythmia or conduction disorder, *n* = 7) were present among 13 individuals, of which 4 also had atrial fibrillation. Valvular heart disease was found among 16 individuals, of which 8 had aortic insufficiency, 3 aortic stenosis, 2 both aortic stenosis and insufficiency, and 3 mitral insufficiency (Supplementary Table 1). Any arrhythmia and/or valvular heart disease were present among 51 individuals, 7 women, and 44 men.

Among patients with atherosclerotic CVD (*n* = 57) half (*n* = 29) had more than one of the included atherosclerotic CVD conditions. The most frequent diagnoses were myocardial infarction (*n* = 25), congestive heart disease (*n* = 20), angina pectoris (*n* = 17), and unstable angina (*n* = 10) (Supplementary Table 1). Eleven individuals had suffered from a cerebrovascular disease, stroke or transient ischemic attack. Among the patients with congestive heart disease, 9 had no registered ischemic heart disease or cerebrovascular disease, but 5 had a diagnosis of hypertension. Cardiovascular disease was observed as the underlying cause of death for 8 of the 23 patients who had died during the evaluation period.

One or several fractures were registered among 85 patients with AS, 26 spinal fractures and 70 non-spinal fractures. Non-spinal fractures most frequently affected the forearm, *n* = 17; the lower leg or ankle, *n* = 13; the hip, *n* = 12; ribs; *n* = 9, the clavicle, *n* = 8, or fingers or toes, *n* = 6. Most spinal fractures were stable compression fractures of the thoracic or lumbar spine (*n* = 15), but one patient had an unstable thoracic fracture requiring surgery. Nine patients suffered from cervical fractures, which for one patient was the underlying cause of death.

A diagnosis of OSAS was observed for 30 patients, mostly men (*n* = 28) (Supplementary Table 1).

### Associations between disease characteristics and the main comorbidities of interest

Higher age increased the probability of all four groups of comorbidities of interest (Table 2). Male sex was associated with a 5-fold risk of OSAS and with a 3-fold increased risk of arrhythmia and/or valvular heart disease (Table 2). Debut of AS at an earlier age, and longer disease duration, respectively, were associated with a higher prevalence of arrhythmia and/or valvular heart disease (*p* = 0.036, Table 2). Ever treatment with NSAID was associated with a lower prevalence of arrhythmia and/or valvular heart disease (Table 2). No statistically significant associations between disease characteristics and atherosclerotic CVD, or spinal or non-spinal fracture, respectively, were noted (Table 2).

**Table 2:** Associations between ankylosing spondylitis disease characteristics and selected comorbidities. Results from simple logistic regression models adjusted for age at the end of the evaluation period and/or sex (as appropriate)

	A Arrhythmia and/or valvular heart disease (*n* = 51)			B Atherosclerotic CVD (*n* = 57)			C Spinal or non-spinal fracture (*n* = 85)			D Obstructive sleep apnoea syndrome (*n* = 30)		
	OR	(95% CI)	*p*	OR	(95% CI)	*p*	OR	(95% CI)	*p*	OR	(95% CI)	*p*
Male sex*	2.57	(1.08; 6.10)	0.033	1.18	(0.53; 2.65)	0.691	1.23	(0.68; 2.21)	0.493	5.31	(1.23; 22.9)	0.025
Age, years*	1.07	(1.04; 1.10)	< 0.001	1.17	(1.12; 1.22)	< 0.001	1.02	(1.01; 1.04)	0.009	1.03	(1.01; 1.06)	0.018
Age at symptom onset, years	0.96	(0.92; 1.00)	0.036	1.01	(0.98; 1.05)	0.457	0.98	(0.95; 1.01)	0.219	1.02	(0.98; 1.06)	0.428
Disease duration, years	1.05	(1.00; 1.09)	0.036	0.99	(0.95; 1.02)	0.457	1.02	(0.99; 1.05)	0.219	0.98	(0.94; 1.03)	0.428
Peripheral arthritis	0.72	(0.38; 1.36)	0.312	0.61	(0.30; 1.23)	0.163	0.92	(0.56; 1.51)	0.727	1.15	(0.53; 2.47)	0.727
Enthesitis	0.67	(0.35; 1.26)	0.211	0.58	(0.28; 1.17)	0.129	1.00	(0.62; 1.65)	0.997	1.12	(0.52; 2.41)	0.778
Anterior uveitis	0.58	(0.30; 1.15)	0.120	1.23	(0.61; 2.48)	0.572	1.12	(0.68; 1.86)	0.650	0.77	(0.34; 1.72)	0.519
Inflammatory bowel disease	1.07	(0.33; 3.48)	0.905	0.83	(0.23; 3.02)	0.783	1.51	(0.62; 3.71)	0.366	0.42	(0.05; 3.32)	0.413
Psoriasis	2.07	(0.49; 8.76)	0.322	0.94	(0.15; 5.88)	0.944	1.06	(0.27; 4.13)	0.935	0.97	(0.12; 8.18)	0.981
csDMARD treatment ever	0.56	(0.27; 1.16)	0.119	1.53	(0.72; 3.25)	0.264	0.98	(0.58; 1.65)	0.947	1.08	(0.49; 2.42)	0.843
TNFi treatment ever	0.56	(0.16; 1.98)	0.372	0.64	(0.14; 3.00)	0.569	0.78	(0.35; 1.72)	0.534	0.81	(0.23; 2.92)	0.747
Glucocorticoid treatment ever	0.78	(0.39; 1.57)	0.484	1.29	(0.61; 2.71)	0.504	1.05	(0.62; 1.79)	0.853	0.62	(0.25; 1.51)	0.290
NSAID treatment ever	0.37	(0.16; 0.85)	0.019	0.98	(0.37; 2.63)	0.970	0.82	(0.37; 1.82)	0.626	0.73	(0.24; 2.21)	0.583
Spinal mobility	0.94	(0.78; 1.13)	0.510	0.93	(0.75; 1.15)	0.510	1.02	(0.87; 1.18)	0.848	0.82	(0.64; 1.07)	0.142
Mean of CRP	0.99	(0.96; 1.03)	0.687	1.02	(0.99; 1.05)	0.140	1.01	(0.99; 1.04)	0.222	0.98	(0.94; 1.02)	0.399

*OR* odds ratio, *CI* confidence interval, *CVD* cardiovascular disease, *AS* ankylosing spondylitis, *csDMARD* conventional synthetic disease modifying antirheumatic drug, *TNFi* tumour necrosis factor inhibitor, *CRP* C-reactive protein

*Model for male sex was adjusted for age at the end of the evaluation period. Model for age was adjusted for sex

### Patterns of comorbidities in AS patients

**Fig. 1:**
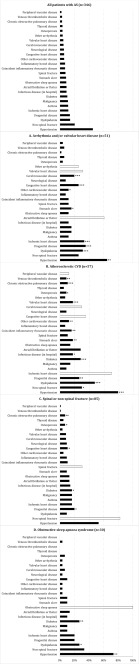
Panel of patterns of comorbidities in patients with ankylosing spondylitis (*n* = 346) overall, and patients with ankylosing spondylitis and the specified comorbidities: (**a**) Arrhythmia and/or valvular heart disease, (**b**) atherosclerotic CVD, (**c**) spinal or non-spinal fracture, or (**d**) obstructive sleep apnoea syndrome. Statistically significant differences in the proportion of a comorbidity compared with individuals without the specified (A–D) comorbidity are marked: * for *p* < 0.05, ** for *p* < 0.01, and *** for *p* < 0.001

Hypertension was the most frequent comorbidity observed in the AS population, affecting 156 (45.1%) individuals (Fig. 1, Supplementary Table 1). More than 10% of the included patients had diabetes, malignancy, asthma, ischemic heart disease, urogenital disease, dyslipidaemia, or non-spinal fracture, respectively (Fig. 1, Supplementary Table 1).

Patients suffering from arrhythmia and/or valvular heart disease had a higher proportion of most other CVD including hypertension, as well as stomach ulcer, urogenital disease, and dyslipidaemia compared with individuals without arrhythmia and/or valvular heart disease (Fig. 1). Most registered comorbidities, including osteoporosis and hospitalisation due to infections, were more frequent among patients with atherosclerotic CVD than among patient with AS not suffering from atherosclerotic CVD (Fig. 1). Prevalence of spinal or non-spinal fracture was related to the presence of chronic obstructive pulmonary disease, osteoporosis, congestive heart disease, diabetes, and urogenital disease (Fig. 1). Patients with OSAS had a higher proportion of features of the metabolic syndrome—diabetes, dyslipidaemia, and hypertension (Fig. 1).

## Discussion

In this cross-sectional study, exploring patterns of comorbidity in patients with established AS, we observed CVD, arrhythmias and valvular heart disease, fractures and OSAS to be associated with age, and arrhythmias and valvular heart disease to be associated with long disease duration, and low age at AS disease onset. Atherosclerotic CVD was linked to numerous other comorbidities, and arrhythmia and/or valvular heart disease was associated mainly with other CVD and CVD-risk factors. Obstructive sleep apnoea syndrome was linked to features of the metabolic syndrome, but no clear pattern was observed for the group of spinal and non-spinal fractures.

Progressive fibrotic changes in the aortic root leading to impaired aortic valve function and conduction abnormalities are frequently observed in patients with AS and can be seen as an extra-articular disease manifestation [6]. The background of CV comorbidity in patients with AS is complex. Disease-specific changes, such as fibrosis, as well as systemic inflammation and traditional CV-risk factors might affect the risk of valvular or congestive heart disease, arrhythmias, and coronary disease [5, 19, 20]. In the present study, we observed a relationship between arrhythmias and/or valvular heart disease and atherosclerotic CVD, which are taken to reflect this complexity. As expected, increasing age was associated with all four groups, but had the largest impact on the group with atherosclerotic CVD. This is also likely to explain why this group was associated with the most other comorbid conditions. The observed association between age or disease duration, respectively, and arrhythmias and/or valvular heart disease are in line with previous studies on conduction disorders or aortic valve disease [5, 7, 21, 22]. In contrast to the present study, some earlier studies have shown associations with measurements of disease severity or AS disease phenotype [7, 21]. None of the DMARD, TNFi or glucocorticoid treatments (which may be suggested as proxies for a more severe disease) or the mean CRP, or spinal mobility, showed any associations with the comorbidities of interest. A proper evaluation of any impact of the disease activity on the development of comorbidities would however require a prospective study design, and preferably an inception cohort. The negative association between NSAID treatment and arrhythmias and/or valvular heart disease is likely due to confounding by contra-indication. We could observe that among the individuals who never had used NSAID, one in three used or had used anticoagulants, which constitute a contraindication for NSAID use.

Despite the high prevalence of fractures in the population, only 3.5% of the patients overall and 7% of the patients with fractures had a diagnosis of osteoporosis. This could reflect insufficient awareness of the risk of osteoporosis in AS, and possibly under-treatment of the condition.

Obstructive sleep apnoea has been suggested as a part of the metabolic syndrome, and also to contribute to the metabolic derangements [23], which could be a background for the associations observed in the present study.

The relatively low percentage of patients ever exposed to TNFi, 14%, can be attributed to the specific setting of the study. A lower uptake of biological drugs in Västerbotten County due to local treatment traditions [24] is one explanation. The second is the inclusion of also patients with a mild AS disease in the study, resulting in a greater denominator compared with other hospital-based AS populations.

Despite of the strengths of this study in terms of generalisability due to the relatively large patient population, the inclusion of patients with a mild as well as severe disease and the available patient records from most medical specialities, there are several limitations needed to take into consideration. Firstly, the included data was retrieved from clinical visits and not from structured follow-ups, which mean that choices to measure or register a variable or not is likely to be influenced by factors related to the health care providers and to characteristics of the patients. An example is the body weight (a parameter not included in the analyses), that was registered for 80% of the patients with OSAS, but only for 40% of the patients without this comorbidity. Secondly, the comorbidities were retrieved from records and any formal validation could not be performed. Some diagnoses of comorbidities were imprecise, such as some arrhythmias or conduction system disorders. It is also likely that comorbidities considered being of minor clinical significance, such as first degree atrioventricular block, less frequently were listed as a diagnosis in the records overview, resulting in a low sensitivity for such comorbidities in the present study. The recordings of disease-specific factors, comorbidities, and events taking place at a time point before the digitization of the medical records might not be complete. The specific selection of patients due to the use of the modified New York Criteria should also be noted as a potential limitation. Due to the explorative nature of the study and the lack of precision in several measurements, we refrained from any statistical handling of missing data. The cross-sectional design does not allow any suggestions of prediction or causality. Thus, the present study has its main value as explorative and hypothesis generating, and the results might not be applicable to another population of patients with AS.

In summary, multiple coexisting comorbidities were frequent in AS, especially among patients with CVD. High age was associated with comorbidity, but among disease characteristics only long disease duration and early disease onset seem to contribute to the risk of comorbidity. The risk of comorbidity in AS motivate clinical awareness. Longitudinal studies are needed to identify predictors and measures to prevent comorbidity in the AS population.

## Electronic supplementary material

ESM 1 (DOCX 141 kb)
