# Supplementary material for: Patterns of comorbidity and disease characteristics among patients with ankylosing spondylitis—a cross-sectional study
Source: Clin Rheumatol. 2017 Nov 8;37(3):647–53. doi: 10.1007/s10067-017-3894-0 (PMC5835056; doi:10.1007/s10067-017-3894-0)
Supplement: Supplementary file 1 — (DOCX 141 kb) [file 10067_2017_3894_MOESM1_ESM.docx]

Supplementary table 1. Numbers of patients (%) with ankylosing spondylitis and each registered comorbidity. An individual can suffer from more than one comorbidity. Comorbidity not included in Figure 1 marked in italic.

|  | Total | | Men | | Women | |
| --- | --- | --- | --- | --- | --- | --- |
| Myocardial infarction | 25 | (7.2) | 22 | (8.4) | 3 | (3.5) |
| Unstable angina | 10 | (2.9) | 8 | (3.1) | 2 | (2.4) |
| Chronic ischemic heart disease | 4 | (1.2) | 2 | (0.8) | 2 | (2.4) |
| Angina pectoris | 17 | (4.9) | 14 | (5.4) | 3 | (3.5) |
| Ischemic stroke | 8 | (2.3) | 5 | (1.9) | 3 | (3.5) |
| Haemorrhagic stroke | 1 | (0.3) | 1 | (0.4) | 0 | (0) |
| Stroke, not specified | 3 | (0.9) | 2 | (0.8) | 1 | (1.2) |
| Transient ischemic attack | 8 | (2.3) | 6 | (2.3) | 2 | (2.4) |
| Peripheral vascular disease | 7 | (2) | 5 | (1.9) | 2 | (2.4) |
| Congestive heart disease | 20 | (5.8) | 15 | (5.7) | 5 | (5.9) |
| Hypertension | 156 | (45.1) | 123 | (47.1) | 33 | (38.8) |
| Atrial fibrillation or flutter | 31 | (9) | 27 | (10.3) | 4 | (4.7) |
| Other arrhythmias or conduction disorders | 13 | (3.8) | 12 | (4.6) | 1 | (1.2) |
| Aortic insufficiency | 10 | (2.9) | 9 | (3.4) | 1 | (1.2) |
| Aortic stenosis | 5 | (1.4) | 4 | (1.5) | 1 | (1.2) |
| Mitral insufficiency | 3 | (0.9) | 2 | (0.8) | 1 | (1.2) |
| Mitral stenosis | 0 | (0) | 0 | (0) | 0 | (0) |
| Venous thromboembolic disease | 10 | (2.9) | 6 | (2.3) | 4 | (4.7) |
| Other cardiovascular disease | 20 | (5.8) | 13 | (5) | 7 | (8.2) |
| Diabetes | 35 | (10.1) | 30 | (11.5) | 5 | (5.9) |
| Dyslipidaemia | 48 | (13.9) | 40 | (15.3) | 8 | (9.4) |
| Thyroid disease | 12 | (3.5) | 4 | (1.5) | 8 | (9.4) |
| *Other metabolic or endocrine disease* | *9* | *(2.6)* | *6* | *(2.3)* | *3* | *(3.5)* |
| Mb Crohn | 7 | (2) | 6 | (2.3) | 1 | (1.2) |
| Ulcerous colitis | 13 | (3.8) | 8 | (3.1) | 5 | (5.9) |
| Other inflammatory gastrointestinal disease | 6 | (1.7) | 4 | (1.5) | 2 | (2.4) |
| Stomach ulcer | 29 | (8.4) | 19 | (7.3) | 10 | (11.8) |
| *Autoimmune ocular disease (not uveitis)* | *3* | *(0.9)* | *3* | *(1.1)* | *0* | *(0)* |
| *Dermatological disease (not psoriasis)* | *9* | *(2.6)* | *7* | *(2.7)* | *2* | *(2.4)* |
| Asthma | 39 | (11.3) | 30 | (11.5) | 9 | (10.6) |
| Chronic obstructive pulmonary disease | 10 | (2.9) | 9 | (3.4) | 1 | (1.2) |
| Obstructive sleep apnoea | 30 | (8.7) | 28 | (10.7) | 2 | (2.4) |
| Other pulmonary disease | 8 | (2.3) | 8 | (3.1) | 0 | (0) |
| Neurological disease | 19 | (5.5) | 10 | (3.8) | 9 | (10.6) |
| Malignancy | 38 | (11) | 29 | (11.1) | 9 | (10.6) |
| Osteoporosis | 12 | (3.5) | 9 | (3.4) | 3 | (3.5) |
| Non-spinal fracture | 70 | (20.2) | 54 | (20.7) | 16 | (18.8) |
| Spinal fracture | 26 | (7.5) | 21 | (8) | 5 | (5.9) |
| Infectious disease (in hospital) | 31 | (9) | 25 | (9.6) | 6 | (7.1) |
| Prostate hyperplasia | 29 | (8.4) | 29 | (11.1) | 0 | (0) |
| Prostatitis | 13 | (3.8) | 13 | (5) | 0 | (0) |
| Urethritis | 3 | (0.9) | 2 | (0.8) | 1 | (1.2) |
| Other urogenital disease | 2 | (0.6) | 0 | (0) | 2 | (2.4) |
| *Chronic kidney disease* | *5* | *(1.4)* | *4* | *(1.5)* | *1* | *(1.2)* |
| Coincident inflammatory rheumatic disease | 24 | (6.9) | 16 | (6.1) | 8 | (9.4) |

Supplementary table 2. Specifications of groups and subgroups of diagnoses of comorbidity collected from patient records

| **Atherosclerotic cardiovascular disease** | | |
| --- | --- | --- |
|  | Ischemic heart disease | |
|  |  | Myocardial infarction |
|  |  | Unstable angina |
|  |  | CABG |
|  |  | PTCA |
|  |  | Chronic ischemic heart disease |
|  |  | Stable angina |
|  | Cerebrovascular disease | |
|  |  | Ischemic stroke |
|  |  | Haemorrhagic stroke |
|  |  | Stroke, not specified |
|  |  | Transient ischemic attack |
|  | Peripheral vascular disease | |
|  | Congestive heart disease | |
| **Arrhythmia and/or valvular heart disease** | | |
|  | Atrial fibrillation or flutter | |
|  | Other arrhythmias or conduction disorders | |
|  | Valvular heart disease | |
|  |  | Aortic insufficiency |
|  |  | Aortic stenosis |
|  |  | Mitral insufficiency |
|  |  | (Mitral stenosis) |
| **Inflammatory bowel disease** | | |
|  | Mb Crohn | |
|  | Ulcerous colitis | |
|  | Other inflammatory gastrointestinal disease | |
| **Spinal or non-spinal fracture** | | |
|  | Non-spinal fracture | |
|  | Spinal fracture | |
| **Urogenital disease** | | |
|  | Prostate hyperplasia | |
|  | Prostatitis | |
|  | Urethritis | |
|  | Other urogenital disease | |
